# Pleiotrophin in Mammary Gland Development and Breast Cancer: A Comprehensive Review of the Evidence

**DOI:** 10.3390/cells15100927

**Published:** 2026-05-18

**Authors:** Arianna S. Gholami, Ciara N. Walsh, Jean McBryan

**Affiliations:** 1 School of Medicine, RCSI University of Medicine and Health Sciences, 123 St Stephen’s Green, D02 YN77 Dublin, Ireland; 2 Department Surgery, RCSI University of Medicine and Health Sciences, Beaumont Hospital, D09 YD60 Dublin, Ireland

**Keywords:** pleiotrophin, breast cancer, transcriptomics, serum, biomarker, drug resistance, angiogenesis, development

## Abstract

**Highlights:**

**What are the main findings?**

- The growth factor PTN signals in a paracrine manner, with RNA and protein often detected in different cell types of the mammary and tumour microenvironments.
- PTN’s functional role is context-dependent; it is not oncogenic in normal mammary development but becomes pro-tumorigenic in breast cancer, with the strongest effects in aggressive subtypes.

**What are the implications of the main findings?**

- The PTN signalling axis represents a therapeutic opportunity, particularly in aggressive subtypes such as triple negative breast cancer and highlights the need for integrated tissue-stromal-circulating analysis in biomarker and drug-development efforts.

**Abstract:**

Pleiotrophin (PTN), a heparin-binding growth factor with potent mitogenic and angiogenic activity, has emerged as a key regulator of mammary gland biology and a potential driver of breast cancer progression. This review integrates current evidence on PTN’s roles from normal mammary development, where it can delay ductal outgrowth, to triple negative breast cancer, where it promotes lung metastasis and correlates with poor survival. Though frequently reported as being overexpressed in breast cancer, the published data indicates that *PTN* transcription is reduced in cancer relative to normal breast cells. By contrast, serum PTN protein levels have been shown by multiple studies to be elevated in breast cancer patients relative to healthy controls. We examine the expression and function of PTN at a cellular level and explore the interplay between PTN and the tumour microenvironment. We evaluate preclinical models, clinical correlations, and emerging biomarker data that position PTN as a candidate prognostic indicator and therapeutic target. Despite growing interest, significant gaps remain regarding context-specific signalling. By integrating developmental and oncogenic perspectives, this review highlights PTN as a pivotal but underexplored factor in mammary gland physiology and breast cancer and outlines future research directions needed to translate PTN-targeted strategies into clinical benefit.

## 1. Introduction

The growth factor pleiotrophin (PTN) has been implicated in diverse developmental and pathological processes, yet its specific relevance to breast cancer remains poorly defined. Although PTN has been studied for more than three decades, the literature on its role in mammary gland biology and tumour progression is fragmented, with inconsistent reports on its expression patterns and context-dependent signalling.

As a secreted protein, PTN has recently been detected in serum of breast cancer patients, where elevated levels correlate with poor overall survival, raising interest in PTN as a potential liquid biomarker [1,2]. PTN has also been implicated in establishing a pro-metastatic niche and in reducing sensitivity to breast cancer therapies [3]. However, no unified framework currently integrates PTN’s developmental functions with its potential oncogenic activities in breast cancer. Uncertainty persists regarding whether PTN expression is truly elevated in breast cancer tissue, and discrepancies exist between RNA and protein expression patterns and between tissue and serum measurements.

This review synthesises current knowledge on PTN across normal mammary biology and breast cancer, clarifying discrepancies in reported expression, examining functional evidence from preclinical models, and evaluating its potential as a biomarker and therapeutic target. By bringing these strands together, we aim to define PTN’s emerging significance and highlight key gaps that must be addressed to advance PTN-directed research.

## 2. Background

Pleiotrophin (PTN) is an 18 kDa secreted protein which acts as a growth factor and cytokine. It is encoded by the PTN gene located on human chromosome 7 (band 7q33) [4]. The human PTN gene spans 116 kb in size, and the protein it encodes plays a multifaceted role in the human body.

PTN was first isolated more than 30 years ago and was initially classified as a member of the fibroblast growth factor (FGF) family due to its ability to stimulate fibroblasts and epithelial cells [5,6,7]. It has since been referred to as various names, including heparin-binding growth factor 8 (HBGF-8), heparin affinity regulatory protein (HARP) and osteoblast-specific factor (OSF-1) [8,9,10]. The name ‘pleiotrophin’ was assigned in 1990 to reflect the varied roles of PTN: the same protein purified from bovine uterus for its mitogenic activity was also identified in new-born rat brain as a factor promoting neurite outgrowth [11].

Since the 1990s, PTN’s known functions have expanded to include roles in angiogenesis [12], synaptic plasticity and neuroprotection [13,14], inflammation and wound repair [15], metabolism [16] and stem cell maintenance [17]. PTN is strongly expressed during embryonic development, with expression decreasing in most tissues following birth. In adults, PTN expression is highest in endocrine glands and the brain [18]. As with many developmentally regulated genes, PTN is highly conserved across species [11].

PTN and the related neurotrophic cytokine midkine (MDK) form a distinct two-member family of heparin-binding growth factors, also known as the pleiotrophin gene family [11,19,20,21,22,23]. MDK, discovered in 1988 in mouse embryonal carcinoma cells, plays key roles in embryonic development [24]. PTN and MDK share approximately 50% amino acid identity, similar domain structures and comparable binding affinity to heparin, glycoproteins and proteoglycans [25]. Although once thought to be functionally redundant, genetic studies show that PTN can compensate for MDK loss but not vice versa, indicating the compensation is not mutual [26].

PTN’s signalling is complex and involves multiple receptors and co-factors. PTN binds to cell-surface proteoglycans containing the polysaccharide glycosaminoglycan (GAG), including syndecans (SDC3), and receptor protein tyrosine phosphatase beta/zeta (PTPRZ1) [12]. It has been demonstrated that PTN inactivates the catalytic phosphatase activity of PTPRZ1 by inducing receptor dimerization or oligomerization, which results in increased beta-catenin phosphorylation, dissociation from E-cadherin and cytoplasmic accumulation [27]. PTN also interacts with nucleolin, neuropilin-1 (NRP-1) [28], integrins (alpha V-beta 3 and alpha M-beta 2), and the anaplastic lymphoma kinase (ALK) receptor [29,30,31]. The complexity of PTN receptor binding has been reviewed elsewhere and will not be covered in detail in this manuscript [29].

The diverse signalling pathways and biological functions attributed to PTN highlight its potential to influence tissue organisation and disease. To understand how these properties become relevant in breast cancer, it is necessary to first define PTN’s expression landscape within the mammary gland.

## 3. PTN Expression in Mammary Tissue and Breast Cancer

### 3.1. PTN Expression During Mammary Gland Development

**Figure 1:**
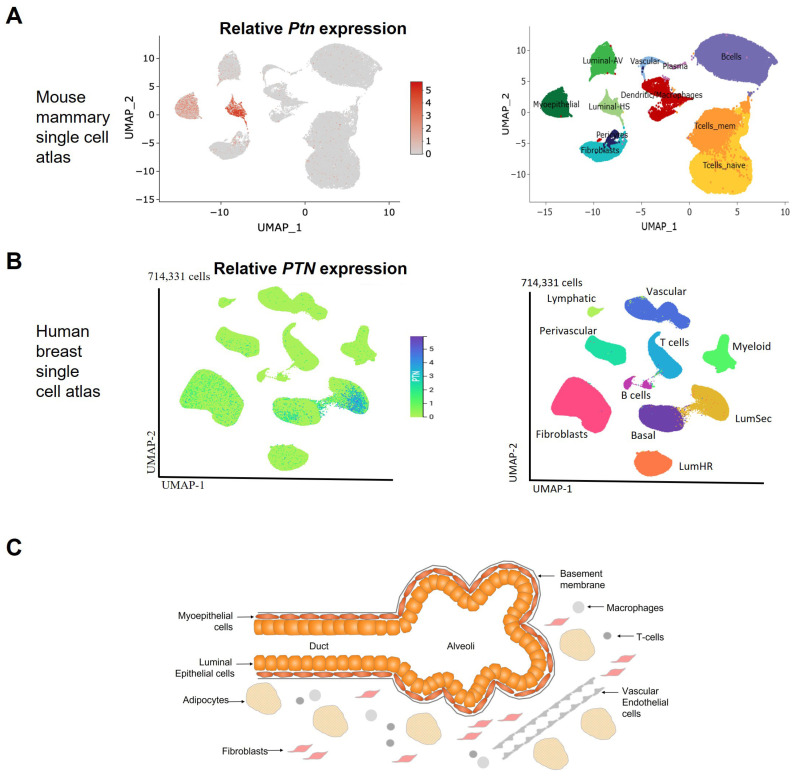
Spatial gene expression of Pleiotrophin in the developing mouse mammary gland and healthy human breast. (**A**). Feature UMAP plot of *Ptn* gene expression across scRNA-seq clusters from the comprehensive single cell ageing atlas of mammary tissues (https://mga.jax.org/, accessed on 18 July 2025) (https://creativecommons.org/licenses/by-nc-nd/4.0/, accessed on 18 July 2025) [34]. Comparison with the reference plot shows that *Ptn* expression is predominantly detected in hormone-sensing luminal epithelial and myoepithelial cells. (**B**). UMAP plot of human *PTN* gene expression across scRNA-seq clusters from The Human Breast Cell Atlas (https://cellxgene.cziscience.com/e/842c6f5d-4a94-4eef-8510-8c792d1124bc.cxg/, accessed on 8 August 2025) [37,38]. Comparison with the reference plot shows that *PTN* expression is predominantly detected in luminal secretory cells and basal-myoepithelial cells. (**C**). Schematic overview of the different cell types of the mammary gland with those expressing *PTN* RNA highlighted in red and orange. Strongest expression has been reported in epithelial and myoepithelial cells of the mammary gland with some *PTN* expression also detected in adipocytes and fibroblasts, but minimal expression reported in endothelial cells or immune cells such as T-cells or macrophages. UMAP: Uniform Manifold Approximation and Projection, Luminal-AV: luminal alveolar, Luminal-HS: luminal hormone sensing, Tcells_mem: memory T cells, Tcells_naive: naïve T cells, Lumsec: luminal secretory cells, LumHR: luminal hormone responsive cells.

The strongest, most reproducible evidence shows that *Ptn* expression in the mouse mammary gland is predominantly located in epithelial cells. In 5-week-old pubertal mice, in situ hybridisation demonstrated expression of *Ptn* RNA in epithelial cells of terminal end buds (TEBs) and mature ducts [32]. Tissue fractionation of virgin mouse mammary glands also confirmed the presence of *Ptn* RNA in the epithelial fraction but not the stromal fraction by Northern blotting [33]. Single cell RNA sequencing (scRNA-seq) has since produced a mouse mammary gland atlas which validated this expression pattern, detecting *Ptn* RNA in epithelial and myoepithelial cells, with minimal expression in stromal, vascular or immune cells of the gland (Figure 1A) [34]. One RT-PCR study also detected strong expression of *Ptn* RNA in adipocytes isolated from 7-week-old mouse mammary glands [35]; adipocytes were not included in the single cell mouse mammary gland atlas due to lysis-based preparation methods, explaining why this had not previously been detected in the atlas [34]. Protein localisation of PTN appears broader than RNA localisation: immunohistochemistry detected PTN in both ductal epithelium and surrounding stroma [33]. Piecing the data together, it is likely that this reflects protein secretion and local retention rather than stromal transcription.

In human breast tissue, early in situ hybridisation studies reported *PTN* RNA predominantly in alveolar myoepithelial cells, with little or no signal in luminal epithelial cells [36]. *PTN* RNA was also detected in vascular structures. These studies were conducted in reduction mammoplasty tissue and in normal tissue opposite to adenocarcinoma. Immunocytochemistry on the same tissues identified PTN protein in capillaries, arterioles, myoepithelial and epithelial cells, suggesting secretion and diffusion from cells of origin [36]. More recent single cell sequencing from the Human Breast Cell Atlas shows *PTN* RNA is predominantly expressed in both luminal epithelial cells and myoepithelial cells (Figure 1B) [37,38], aligning more closely with mouse data. Some species-specific differences remain—mouse *Ptn* is reportedly enriched in hormone-sensing epithelia, whereas human *PTN* is enriched in luminal secretory cells—but methodological differences in single cell analysis likely contribute to this.

PTN protein is not confined to the cells that transcribe it. Supporting its secretory nature, PTN has been detected in human breast milk, with higher levels in colostrum than mature milk [39]. Overall, as summarised in Figure 1C, the available data indicate that *PTN* is transcribed in epithelial and myoepithelial compartments in the mammary gland. Adipocytes may express *PTN*, but the magnitude relative to epithelial cells is unknown. PTN protein is detectable more broadly due to secretion and diffusion.

**Figure 2:**
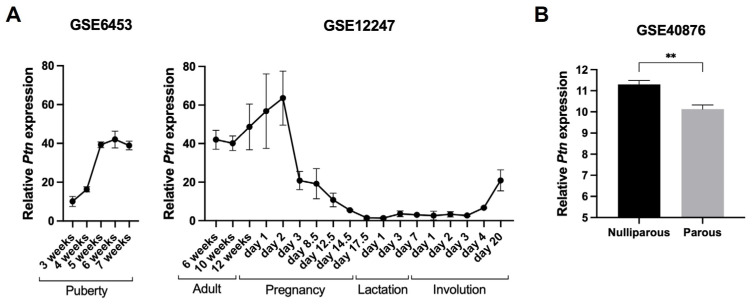
Temporal gene expression of Pleiotrophin in the developing mouse mammary gland. (**A**) Line graphs display the relative expression of *Ptn* RNA over the time course of mouse mammary gland development. Data from publicly available microarray datasets GSE6453 (probe ID 1448254_at, *n* = 3) [40] and GSE12247 (probe ID 97474_r_at, *n* = 3) [42]. (**B**) Bar graph shows the relative expression of *Ptn* RNA between nulliparous and parous mouse mammary glands. Data from publicly available microarray dataset GSE40876 (probe ID 10543959, *n* = 4) [43]. ** *p* < 0.01, unpaired t-test.

Temporally, *Ptn* RNA is highest in the virgin mouse mammary gland, with expression rising sharply during puberty [40,41]. Expression remains elevated during the first 10 days of pregnancy but then falls 30-fold by day 15 of pregnancy as the gland transitions from proliferation to lobuloalveolar development [33,41]. *Ptn* expression remains low during lactation and then climbs gradually during involution (Figure 2A) [42]. Parity also influences expression: after the first pregnancy, *Ptn* levels can return to baseline but after multiple pregnancies, expression remains up to 8-fold lower than in nulliparous mice (Figure 2B) [33,43]. This parity-associated downregulation has been proposed to contribute to the protective effect of multiparity against breast cancer.

Together, these datasets indicate that *Ptn* is transcribed in epithelial, myoepithelial and adipocyte compartments, while protein is detectable more broadly due to secretion. Temporally, *Ptn* aligns with proliferative phases of mammary development and is downregulated during differentiation and with increasing parity.

### 3.2. PTN RNA Expression in Breast Cancer

Early studies suggested that *PTN* was frequently expressed in breast cancer. The first report, using RNase protection assays on 27 tumours, detected *PTN* RNA in 62% of samples and noted no detectable expression in adjacent normal tissue [5]. This study has been widely cited as evidence of high *PTN* expression in breast cancer. A subsequent PCR-based study also detected *PTN* RNA in the majority of breast tumour samples examined but found levels comparable to normal tissue [44]. A larger RNase protection assay later reported *PTN* RNA in all 64 breast tumours examined, although the authors noted that some samples had very low levels of detection that might have fallen below detection thresholds in earlier work [45].

**Figure 3:**
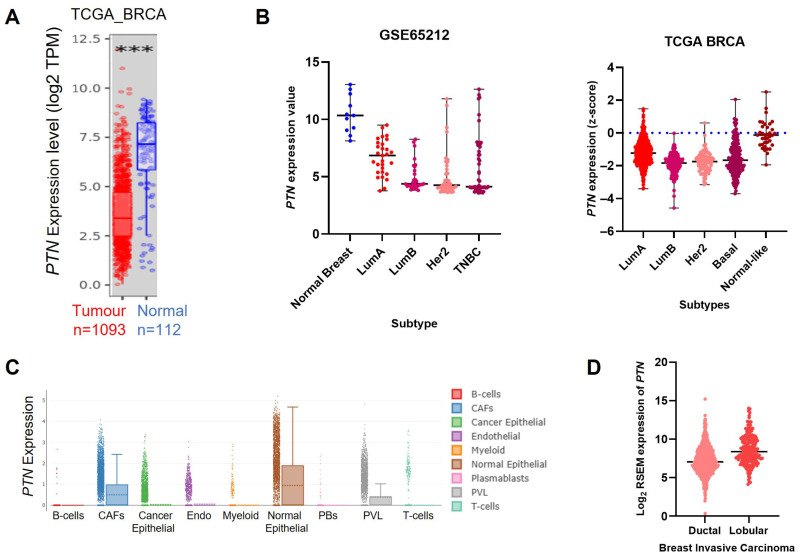
Gene expression of Pleiotrophin in breast cancer. (**A**) Relative expression of *PTN* in 1093 breast tumours and 112 normal breast samples from TCGA PanCancer Atlas. Data from Cistrome website, timer application, http://cistrome.org/TIMER/, accessed on 13 August 2025, *** *p* < 0.001, Wilcoxon test. (**B**) Relative expression of *PTN* in different subtypes of breast cancer, analysed with one-way ANOVA and Tukey’s multiple comparison test. Left graph is Affymetrix microarray data from GSE65212 showing *PTN* expression is significantly higher in normal breast (*n* = 11) compared to luminal A (*n* = 29), luminal B (*n* = 30), Her2+ve (*n* = 39) or triple negative (*n* = 55) breast cancer subtypes (*p* < 0.0001 for each comparison). The luminal A subtype is also significantly higher than luminal B or Her2+ve subtypes (*p* < 0.05). Data from GEO2R https://www.ncbi.nlm.nih.gov/geo/geo2r/, accessed on 13 August 2025 [49]. Right hand graph is RNAseq data from TCGA PanCancer Atlas. *PTN* expression is plotted relative to normal breast tissue which is represented by the blue dotted line. Normal-like breast cancer (*n* = 36) has significantly higher *PTN* than luminal A (*n* = 499), luminal B (*n* = 197), Her2+ve (*n* = 78) or basal (*n* = 171) cancer subtypes (*p* < 0.0001 for each comparison). The luminal A subtype is also significantly higher than luminal B, Her2+ve or basal subtypes (*p* < 0.0001 for each comparison). Basal subtype is significantly higher than luminal B (*p* < 0.001). Data from cbioportal https://www.cbioportal.org/, accessed on 13 August 2025 [47,48]. (**C**). Single cell RNAseq analysis of PTN expression from 100,000 breast cancer cells from all subtypes. Highest *PTN* expression is detected in normal epithelial cells, cancer-associated fibroblasts (CAFs) and perivascular-like phenotype (PVL) cells. Graph produced with https://singlecell.broadinstitute.org/single_cell/study/SCP1039/a-single-cell-and-spatially-resolved-atlas-of-human-breast-cancers/, accessed on 11 February 2026 [50]. (**D**). RNAseq data from TCGA PanCancer Atlas showing higher *PTN* expression in invasive lobular cancer (*n* = 258) compared to invasive ductal breast cancer (*n* = 837) (*p* < 0.0001, unpaired *t*-test). Data from cbioportal https://www.cbioportal.org/, accessed on 13 August 2025 [47,48].

With the explosion of gene expression microarray and RNA sequencing technologies, *PTN* RNA levels have now been measured in thousands of breast tumours, providing a clearer picture. Although “normal” tissue varies across studies—ranging from normal regions within a tumour sample, to adjacent normal regions, to healthy donor breast—modern datasets consistently show that *PTN* RNA is lower in breast tumours than in normal breast tissue. In The Cancer Genome Atlas (TCGA), which includes >1000 primary breast tumours and >100 adjacent normal samples, *PTN* expression is significantly reduced in tumour tissue (Figure 3A,B) [46,47,48]. A microarray study comparing 130 tumours with 11 healthy reduction mammoplasty samples similarly found lower *PTN* RNA in tumours (Figure 3B) [49]. Together, these large datasets contradict the conclusions drawn from the original 1992 study and there now exists mounting evidence for reduced *PTN* RNA expression in breast cancer relative to normal breast tissue.

Single cell RNA-sequencing further supports this pattern. In a dataset of 26 primary breast tumours, *PTN* expression was highest in normal epithelial cells within the tissue, with substantially lower expression in cancer epithelial cells (Figure 3C) [50]. Of interest, relatively high *PTN* RNA was detected in cancer associated fibroblasts (Figure 3C). As we proceed to compare bulk *PTN* expression data between different types of breast cancer, it is worth bearing in mind that stromal composition of any tumour sample is likely to impact the overall *PTN* expression levels detected.

Across breast cancer subtypes, *PTN* expression is highest in luminal A tumours compared to luminal B, Her2+ve or basal subtypes. This pattern is evident in both TCGA and the large microarray dataset previously mentioned (Figure 3B) [46,51]. The Pam50 classification of normal-like breast cancer, although rare, shows *PTN* levels comparable to normal breast tissue (Figure 3B) [46,51].

Histological subtype also influences *PTN* expression. Invasive lobular carcinomas (ILC) show higher *PTN* RNA levels than invasive ductal carcinomas (IDC) in TCGA (Figure 3D). This likely reflects the fact that 90% of the ILCs are luminal A or normal-like compared with only 46% of the IDCs [46].

Comparisons between primary and metastatic tumours are more complex. In the AURORA study of 152 matched primary-metastatic pairs, *PTN* expression was lower in metastases (log2 fold change −0.497, *p* = 0.00015) [52]. However, the authors note that many differences reflect the tissue environment rather than tumour-intrinsic changes, and metastatic samples often lack *PTN*-expressing normal breast cells, complicating interpretation.

**Figure 4:**
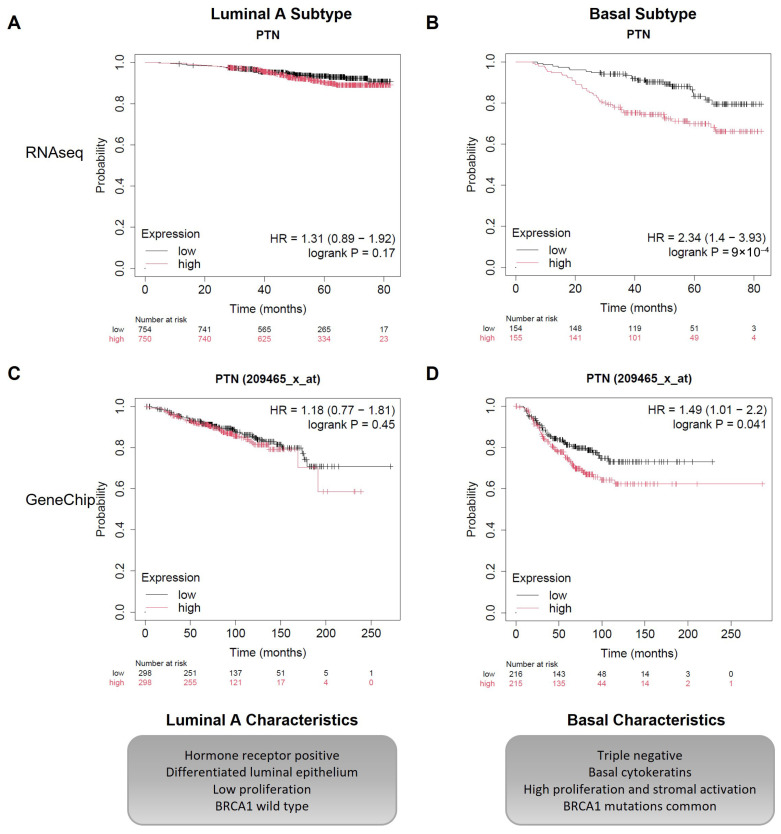
Association between *PTN* expression and overall survival is subtype-specific. Kaplan–Meier curves depict overall survival stratified by median *PTN* expression. (**A**,**C**). No statistically significant difference in overall survival was observed between the high-*PTN* and low-*PTN* expression groups in luminal A breast cancer patients. This was seen in both the TCGA RNAseq dataset (**A**) with *n* = 1504 patients, log-rank *p* = 0.17 and the GeneChip microarray data set (**C**) with *n* = 596 luminal A patients, log-rank *p* = 0.45. (**B**,**D**). Within the basal subtype of breast cancer, patients with high *PTN* expression experienced significantly poorer overall survival compared to those with low expression. This was seen in both the TCGA RNAseq dataset (**B**) with *n* = 309 patients, log-rank *p* = 0.0009 and the GeneChip microarray dataset (**D**) with *n* = 431 patients, log-rank *p* = 0.041. Images were generated using Kaplan–Meier plotter with PAM50 classifications (https://kmplot.com/analysis/index.php?p=home, accessed on 20 August 2025) [53]. Key characteristics of each subtype are highlighted below the Kaplan–Meier plots.

Few studies have examined whether *PTN* RNA correlates with clinical outcome. One analysis of lymph-node-positive breast cancer patients in TCGA reported worse overall survival in patients with high *PTN* expression. Median overall survival was 59.9 months for patients with low *PTN* expression (*n* = 220), versus 33.6 months for those with high *PTN* expression (*n* = 232) [3]. A similar trend was observed in a very small cohort of stage IV patients (*n* = 20) [3]. Using KMPlotter, *PTN* expression showed no association with overall survival in luminal A cancers from either the RNA-seq (*n* = 1504) or microarray (*n* = 596) datasets (Figure 4A,C). In contrast, high *PTN* expression was associated with significantly worse overall survival in the basal subtype of cancers in both RNA-seq (*n* = 309) and microarray (*n* = 431) datasets (Figure 4B,D) [53,54]. A survival association was also observed in HER2 positive cancers in the RNA seq dataset (*n* = 295 Her2+ve patients, *p* = 0.031) but not in the microarray dataset (*n* = 439 Her2+ patients, *p* = 0.54) [53,54].

Overall, *PTN* RNA expression is reduced in breast cancer relative to normal breast tissue, with normal-like and luminal A tumours retaining higher *PTN* levels than more aggressive subtypes. The prognostic significance of *PTN* appears to be subtype-specific: high *PTN* expression is associated with poorer overall survival in basal-like cancers but not in luminal A disease.

### 3.3. PTN Protein Expression in Breast Cancer

Although RNA-based datasets consistently show reduced *PTN* transcript levels in breast cancer relative to normal breast tissue, RNA and protein abundance do not always correlate. This is especially true for secreted proteins such as PTN, whose protein distribution may extend beyond the cells that transcribe it. Given the known differences between PTN RNA and protein localisation in normal mammary tissue, it is important to evaluate PTN protein expression directly in breast cancer.

An immunohistochemical analysis, published in 2016, examined PTN protein in 325 breast tumour samples, 30 breast fibroadenoma and 30 metastasised lymph nodes [55]. The study was made up of 66 luminal A, 67 luminal B, 78 Her2+ve, 78 TNBC and 36 cases with TNBC that relapsed within 3 years (RTNBC). No significant difference in PTN protein levels was detected between different subtypes or between breast cancer and the fibroadenoma controls. Only the relapsed TNBC samples showed significantly higher PTN protein expression, accompanied by increased expression of the PTN receptor PTPRZ1 [55]. Notably, the coefficient of variation within each group was high, limiting statistical power.

A 2017 immunohistochemical study of 80 primary tumours and 80 matched paracancerous samples reported a different pattern. In this study the “normal” regions were sampled approx. 3 cm from the cancers. PTN protein levels were significantly higher in tumour tissue than in adjacent non-malignant regions, with 47/80 cancer samples displaying high PTN staining compared with 27/80 paracancerous regions [1]. Higher PTN protein expression was significantly correlated with advanced TNM stage (*p* = 0.017), positive lymph node status (*p* = 0.043), and poor differentiation status (*p* = 0.026), but not with ER, PR, Her2 status, tumour size or patient age. Although the cohort was not stratified by molecular subtype, the distribution of ER-positive and Her2-positive cases suggests a representative sample.

A third study, also from 2017, measured PTN protein in washouts from core needle biopsies using ELISA (13 malignant, 38 benign samples) [56]. No significant difference in PTN levels was observed between benign and malignant samples, although one LCIS sample displayed markedly elevated PTN (24-fold above the benign mean). Given the small sample size, and the unique nature of the LCIS case, this finding remains preliminary [56].

Across the three studies comparing normal versus cancerous breast tissue, two reported no difference in PTN protein levels, while one reported higher PTN protein in tumours. This contrasts with RNA-based datasets which consistently show higher *PTN* transcript levels in normal breast tissue. Several explanations may account for this discrepancy. Post-transcriptional regulation could suppress PTN translation in normal cells or secretion dynamics could impact the ratio of RNA:protein produced by any given cell. Alternatively, PTN protein detected in cancer cells may originate from adjacent normal cells or the surrounding tumour microenvironment, reflecting PTN’s secreted nature. Methodological factors may also contribute: protein studies involve smaller cohorts (tens to hundreds) compared to the RNA sequencing studies (thousands), and variability in antibody specificity or tumour sampling could influence results, particularly given the heterogenous cellular composition of breast tumours. Improving our understanding of the origin of PTN protein in cancer cells would be beneficial if we are to move towards using PTN as a prognostic or therapeutic target.

A more recent immunohistochemical analysis investigated PTN protein in 58 primary tumours and 36 lymph nodes, without including normal breast controls. Patients with lymph node positive disease displayed stronger PTN staining in both primary tumours and lymph node samples [3]. Multiplex immunohistochemistry in two cases suggested that PTN protein is expressed predominantly by tumour cells, with lower expression in stromal and endothelial compartments—consistent with scRNA-seq findings. The association between high PTN protein and lymph-node positivity aligns with the earlier protein findings of Ma and colleagues, despite differences in antibodies and patient cohorts [1,3].

### 3.4. Circulating PTN Protein Levels in Breast Cancer Patients

Given that PTN is a secreted protein, several groups have examined whether it can be detected in the bloodstream of breast cancer patients and whether circulating levels reflect disease status. The first comprehensive study, by Ma and colleagues, measured serum PTN in 105 breast cancer patients (68 with primary and 37 with metastatic disease) and 40 healthy volunteers using ELISA [1]. Serum PTN levels were markedly higher in patients than in controls (median 471 pg/mL versus 218 pg/mL), mirroring their findings in tumour tissue. Using a cut-off of 399 pg/mL, 72% of patients had elevated PTN, outperforming established tumour markers such as CEA (31%) and CA15-3 (35%). Higher serum PTN was associated with advanced stage, lymph node positivity, poor differentiation and metastatic disease [1]. In a subset of 20 patients, serum PTN levels fell significantly after mastectomy, supporting a breast tumour-associated source, although removal of adjacent normal breast tissue may also have contributed to the fall in PTN serum levels following mastectomy.

A 2023 study extended these observations using plasma from 210 breast cancer patients and 28 healthy individuals [3]. PTN was rarely detectable in healthy controls, whereas patients showed consistently elevated levels. Using a 1 ng/mL threshold, high plasma PTN was associated with significantly worse overall survival, highlighting potential prognostic value.

Similar findings were reported in an Indonesian cohort of 64 patients, where serum PTN levels were substantially higher in metastatic than in non-metastatic disease (mean 43.11 pg/mL vs. 12.53 pg/mL) [2]. Receiver Operating Characteristic (ROC) analysis identified a serum cut-off of 24.7 pg/mL that discriminated metastatic from non-metastatic cases [2].

A 2024 study focussing on inflammatory breast cancer (IBC) also found elevated serum PTN in IBC (*n* = 26) compared with non-IBC patients (*n* = 30) [57]. IBC is a rare and aggressive subtype, and the authors identified a PTN-secreting luminal progenitor population that promoted angiogenesis. Serum PTN levels correlated with PTN protein expression in normal tissue adjacent to IBC, suggesting that tumour–microenvironment interactions may influence circulating PTN [57].

**Figure 5:**
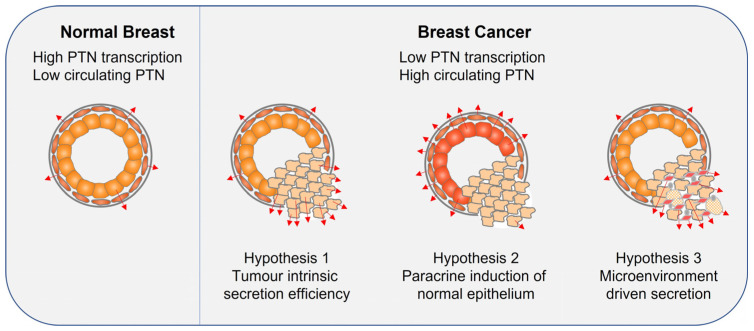
Proposed mechanisms underlying elevated protein levels of PTN in breast cancer despite reduced RNA transcription in tumour cells. The schematic illustrates three non-mutually exclusive hypotheses to explain why blood levels of PTN protein are higher in breast cancer patients even though cancer cells themselves do not show increased transcription of *PTN*. Left panel: Normal breast tissue composed of myoepithelial and luminal epithelial cells, each transcribing baseline amounts of *PTN*, resulting in relatively low circulating PTN levels. Right panel: Breast cancer tissue composed of cancer cells that transcribe lower levels of *PTN* compared to normal breast epithelial cells. Hypothesis 1: Despite reduced transcription in cancer cells, altered secretion dynamics may lead to increased protein production and/or secretion of PTN from the cancer cells. Hypothesis 2: Breast cancer cells growing adjacent to normal epithelial and myoepithelial cells may stimulate these neighbouring non-malignant cells to increase production and/or secretion of the protein, leading to elevated systemic levels without increased tumour cell transcription. Hypothesis 3: In addition to tumour–epithelial interactions, recruitment of additional stromal components into the tumour microenvironment—such as cancer associated fibroblasts (CAFs), adipocytes and other infiltrating cell types—may expand the pool of protein producing cells, thereby contributing to the observed rise in circulating PTN levels.

Across these four studies, a consistent pattern emerges: circulating PTN levels are higher in breast cancer patients than in healthy individuals and tend to increase with disease severity, metastatic spread and poorer prognosis. The clinical appeal is clear—blood sampling is minimally invasive, and PTN may offer diagnostic or prognostic value. However, substantial variability exists in the absolute PTN concentrations reported, with cut off values differing by one to two orders of magnitude despite all studies using ELISA. The cellular origin of circulating PTN also remains unresolved. Bulk RNA sequencing indicates that breast cancer cells do not overexpress *PTN* transcripts, raising the possibility that tumour presence alters PTN production in neighbouring cells or modifies secretion dynamics. Leading hypotheses to reconcile the RNA-protein discrepancy are summarised in Figure 5.

Together, these RNA-protein expression patterns suggest that PTN’s biological activity may not be fully explained by expression patterns in cancer cells alone. The discordance between transcript abundance and protein localisation raises the possibility that the protein is produced or stabilised in a microenvironment compartment distinct from the cells expressing the RNA. This in turn, points towards a model in which paracrine interactions shape the observed phenotypes. These insights set the stage for interpreting the functional studies reviewed in the next Section, particularly in relation to possible microenvironment-driven effects on angiogenesis, migration and invasion.

## 4. Functional Roles of PTN in Mammary Gland Development and Breast Cancer

### 4.1. PTN in Normal Mammary Gland Development

To investigate the function of PTN in the developing mammary gland, PTN knockout mice have been generated. These mice are viable and fertile with no major anatomical abnormalities, and no mammary gland phenotype has been reported [58]. As a result, much of our understanding of PTN’s developmental role comes from studies using a PTN-blocking antibody. Pubertal mice treated with the PTN-blocking antibody showed enhanced epithelial ductal outgrowth with an increase in duct length and number of branch points [33]. Although the number of TEBs was unchanged, treated mice displayed a greater number of terminal ends. These findings led the authors to conclude that endogenous PTN normally delays ductal outgrowth, branching and terminal end formation without altering the final structure of the mature gland [33].

A complementary approach, using a transgenic mouse model that overexpressed PTN under control of the MMTV (mouse mammary tumour virus) promoter, also supported a non-oncogenic role. Despite elevated PTN expression, these mice underwent normal mammary gland development and lactation and did not develop any tumours. Detailed histological analysis revealed no abnormalities [59]. Together, these in vivo models indicate that PTN is not essential for mammary gland development and is not oncogenic on its own.

At the cellular level, primary mammary epithelial cells from 7–9-week-old mice have been cultured in both 2D and 3D systems to assess the function of PTN [33]. Using a PTN blocking antibody, investigators found that PTN did not influence epithelial cell proliferation in either culture condition. Instead, PTN inhibition enhanced cell motility and invasion. In 3D cultures, blocking PTN also increased laminin deposition, leading the authors to propose that PTN disrupts epithelial–extracellular matrix adhesion by reducing laminin deposition, without directly affecting proliferation [33]. In contrast, a co-culture study using immortalised HC-11 mouse mammary epithelial cells with 3T3 fibroblasts reported a pro-proliferative effect of PTN. Of relevance, the immortalised epithelial cells in this system did not express PTN; rather knockdown of PTN in fibroblasts reduced epithelial proliferation, highlighting a stromal-derived, context-dependent effect [35].

Overall, PTN is not required for normal mammary gland development and does not act as an intrinsic oncogene. Instead, PTN appears to transiently restrain ductal outgrowth in vivo and, depending on the cellular and microenvironmental context, can influence epithelial motility, invasion, extracellular matrix interactions, and, under specific conditions, proliferation.

While PTN plays a largely non-essential and tightly regulated role in normal mammary development, its function changes markedly in the malignant setting. This shift becomes evident when PTN is examined in breast cancer models, where it contributes to tumour progression and metastatic behaviour.

### 4.2. PTN in Breast Cancer Progression and Malignancy

Early studies in non-breast cancer systems first suggested a pro-tumorigenic function for PTN. In 1993, PTN was shown to transform mouse 3T3 fibroblasts to a malignant phenotype and enable them to form tumours in nude mice [60]. Conversely, reducing PTN expression in a human melanoma cell line decreased tumour growth, angiogenesis and metastatic spread in xenograft models [61]. These findings prompted investigation into whether PTN similarly contributes to breast cancer aggressiveness.

Evidence for a malignant role of PTN in breast cancer has come from multiple models in which PTN expression or activity was reduced. In the MDA-MB-231 triple negative breast cancer cell line, expression of a dominant-negative PTN mutant abolished the transformed phenotype and eliminated tumour forming ability in athymic nude mice [62]. In parallel, functional inhibition of PTN using a polyclonal anti-PTN antibody reduced proliferation and colony forming capacity of MDA-MB-231 cells in vitro [63]. More recently, the MMTV-PyMT transgenic mouse model has been used to examine PTN function in vivo. Expression of the polyoma middle T oncogene (PyMT) under control of the mouse mammary tumour virus (MMTV) leads to rapid mammary tumour formation of luminal-B-like tumours as well as lung metastases in these mice. PTN-null MMTV-PyMT mice developed tumours more slowly, had smaller tumours at comparable stages, exhibited fewer lung metastases, and showed improved overall survival compared with PTN wild type controls [3]. Together, these studies support a functional role for PTN in sustaining malignant behaviour in both triple negative and luminal B-like breast cancer models.

To tease apart the impact of PTN on the evolution of malignant phenotypes, Ganguly and colleagues then conducted a series of experiments where they could control when PTN was switched off. Moving away from the PTN-null mice, 3B10 was used instead which is a mouse monoclonal antibody that can be delivered at specified times to neutralise PTN. In an orthotopic 4T1 triple negative breast cancer model, treatment with 3B10 after the tumours became palpable did not affect primary tumour growth but significantly reduced lung metastases and improved survival [3]. Similar results were observed in the MMTV-PyMT model when 3B10 treatment was initiated after primary tumour formation. A third model, EO771-LG orthotopic tumours, also showed reduced lung metastatic burden following 3B10 treatment. The EO771-LG cells are a lung metastatic variant of EO771 cells derived from a spontaneous mammary adenocarcinoma in c57black6 mice [3]. Across these models, delayed PTN inhibition consistently reduced lung metastases and extended survival.

The reduction in lung metastases associated with PTN inhibition may reflect impaired seeding or colonisation at secondary sites. Supporting this, pre-treatment of c57black6 mice with 3B10 before tail vein injection of EO771 LG cells resulted in reduced lung metastatic burden [3]. Earlier work by Ducès and colleagues also demonstrated that MDA-MB-231 cells expressing a truncated PTN construct were less tumorigenic when injected subcutaneously into nude mice [63]. Interestingly, prophylactic delivery of the truncated PTN plasmid into mouse muscle before subcutaneous implantation of unmodified MDA-MB-231 cells also reduced tumour formation. This finding is exciting from a clinical perspective because it indicates that altering PTN levels in the surrounding tissue can impair colonisation independently of direct tumour-cell targeting. However, the truncated PTN plasmid used in these studies may also affect the related protein Midkine (MDK), and the authors acknowledged this potential lack of specificity [63].

Collectively, available data indicate that PTN supports metastatic colonisation, particularly in the lung. To date, most functional studies have focused on lung metastasis, and it remains unclear whether PTN similarly influences colonisation of other common breast cancer metastatic sites such as liver, brain, or bone. An in silico analysis has reported PTN as associated with soft tissue (lung and liver) but not bone metastasis in experimental models [64], suggesting potential site-specific roles that warrant further investigation.

Beyond its tumour cell intrinsic effects, PTN also influences the tumour microenvironment, most notably through its ability to promote angiogenesis. This pro-angiogenic activity was one of the earliest malignant functions attributed to PTN.

### 4.3. PTN-Driven Angiogenesis in Breast Cancer

PTN’s pro-angiogenic function in breast cancer was first identified in the 1990s [21]. Choudhuri and colleagues overexpressed PTN in the ER-positive MCF7 breast cancer cell line. In vitro, PTN expression did not alter the monolayer growth of these epithelial cells. However, because MCF7 cells rarely form tumours in xenograft models without additional support, the investigators co-implanted MCF7 cells with either 3T3 fibroblasts or MD-435S cancer cells into oestrogen-supplemented nude mice. Under these conditions, PTN-overexpressing MCF7 cells formed faster-growing tumours than control cells. These tumours also displayed increased vascular density, and BrdU staining showed higher proliferation in both tumour cells and endothelial cells compared with control tumours. This study established PTN as an angiogenic factor in breast tumour development and highlighted the paracrine nature of PTN signalling: PTN overexpression did not enhance proliferation of isolated epithelial cells in vitro but did promote tumour and endothelial proliferation in vivo within a microenvironmental context [21].

Our understanding of PTN-driven angiogenesis largely comes from studies in non-breast cancer disease settings. These have been reviewed elsewhere [29,65], and some evidence suggests that related mechanisms might also be relevant in breast cancer [66]. Of note, it has been suggested that PTN can promote VEGF-independent angiogenesis, that anti-VEGF therapy can induce PTN expression and so PTN may contribute to mechanisms of anti-VEGF therapy resistance [66].

Angiogenesis represents only one aspect of PTN-mediated microenvironmental regulation. PTN also plays a broader role in shaping stromal architecture, influencing fibroblast activation, extracellular matrix composition, and tumour–stroma interactions.

### 4.4. PTN-Mediated Stromal Remodelling

PTN has been shown to remodel the tumour microenvironment in breast cancer. The same group who demonstrated that PTN alone is not oncogenic, also developed three additional models to examine how PTN influences tumour–stroma interactions [59].

In the first model, MMTV-PTN overexpressing mice were crossed with tumour-developing MMTV-PyMT mice. Unlike the reduced tumour progression observed in PTN-null MMTV-PyMT mice [3], PTN overexpression did not significantly alter tumour onset, tumour volume, tumour burden, or metastatic index [59]. The MMTV-PyMT model is known for substantial variability in tumour number and size, which may have limited the ability to detect differences. However, PTN-overexpressing mice displayed a two-fold increase in scirrhous carcinoma foci. These foci were enriched for *Ptn* RNA and contained increased matrix proteins, including collagen and elastin fibrils. A 1.5-fold increase in intratumour microvessel density was also apparent in MMTV-PyMT-PTN mice [59].

The second model used xenografts generated from the ER-positive MCF7 breast cancer cells overexpressing PTN. As expected, subcutaneous injection of MCF7 cells resulted in poor tumour formation. Overexpression of PTN (MCF7-PTN) significantly enhanced tumour-forming capacity, and co-implantation with 3T3 fibroblasts further increased tumour size [59]. The MCF7-PTN xenografts phenotypically resembled the scirrhous carcinoma foci seen in MMTV-PyMT-PTN tumours, forming invasive nodules surrounded by activated fibroblast-like stromal cells, abundant extracellular matrix proteins such as collagen, and extensive neovascularisation [59]. By contrast, control MCF7 tumours, when present, were surrounded by fat vacuoles, fibroblasts and various immune cells, underscoring the ability of PTN-expressing tumour cells to reshape the surrounding microenvironment.

The third model examined interactions between MCF7-PTN cells and 3T3 fibroblasts in vitro. When co-cultured to confluence, MCF7-PTN cells formed distinctive epithelial islands surrounded by acellular regions. This pattern did not occur when wild-type MCF7 cells were co-cultured with 3T3 fibroblasts. Knockdown of PTN using siRNA disrupted epithelial island formation, as did expression of a mutant PTN lacking a secretory signal [59]. These findings indicate that both PTN expression and secretion, together with fibroblast co-culture, are required for the formation of these epithelial islands.

Given PTN’s capacity to remodel the tumour microenvironment and support malignant progression, an important question is whether PTN also influences therapeutic response. Emerging evidence indicates that PTN can modulate sensitivity to chemotherapy, targeted agents, and immunotherapy.

### 4.5. PTN-Mediated Modulation of Drug Response

Chemotherapy can modulate PTN expression. Analysis of patient tumour samples from the PROMIX trial showed that chemotherapy can increase *PTN* and *PTPRZ1* RNA expression. Paired biopsies collected before and after two cycles of epirubicin and docetaxel from 69 patients with Her2-negative, locally advanced breast cancer with no sign of distant metastasis were analysed. The cohort included 21% luminal A, 45% luminal B, 5% Her2-enriched, 21% basal and 8% normal-like tumours. Microarray analysis demonstrated significant increases in *PTN* and *PTPRZ1* expression following chemotherapy [67]. In a pre-clinical model, MDA-MB-231 cells treated with the chemotherapeutic agent doxorubicin also showed increased PTN and PTPRZ1 protein compared to untreated controls [67]. The chemotherapy-induced upregulation formed a positive feedback loop, as doxorubicin-induced PTN expression required PTPRZ1 and vice versa. Addition of recombinant PTN to the culture media of MDA-MB-231 cells counteracted the proliferation-inhibitory effects of doxorubicin and enhanced cell survival. These findings indicate that PTN can promote proliferation, reduce apoptosis, and decrease chemotherapy sensitivity in this triple-negative model [67].

The role of PTN in modulating drug sensitivity in luminal breast cancer has been less extensively studied. PTN was reported to be expressed at higher levels in MCF7/ADR cells than parental MCF7 cells [67]. However, the origin of MCF7/ADR has since been questioned and they may derive from an ovarian cancer line, complicating interpretation of their chemotherapy-resistant phenotype. A 2024 study examined the experimental FAK inhibitor Y15 [68]. In both MCF7 (luminal A) and MDA-MB-231 (triple negative) cells, addition of purified PTN protein increased proliferation and migration in vitro. Treatment with Y15 reduced cell viability and increased apoptosis, but these effects were attenuated when PTN was present. Consistent with this, Y15 treatment following PTN-targeted shRNA transfection produced greater reductions in cell viability and increased apoptosis [68]. These results suggest that PTN can reduce sensitivity of both luminal and triple negative breast cancer cells to FAK inhibition.

PTN has also been implicated in resistance to the anti-angiogenic agent sunitinib. In an orthotopic metastatic breast cancer model using luciferase-expressing 4T1 cells, mice were treated daily with sunitinib beginning seven days after tumour implantation. Animals were classified as untreated, innately resistant, or having acquired resistance to sunitinib. Microarray analysis showed elevated PTN expression in both the innately resistant and acquired resistant tumours [69].

Finally, given the increasing use of immunotherapy in breast cancer, Ganguly and colleagues investigated whether PTN influences response to the PD-1 inhibitor pembrolizumab. To model metastatic disease, mice were tail-vein injected with EO771-LG cells and allowed seven days for micro metastases to establish before treatment. Mice receiving triple therapy with 3B10 (anti-PTN), pembrolizumab (anti–PD-1), and Abraxane (chemotherapy) had significantly reduced lung metastatic burden relative to controls [3]. The triple combination was more effective than 3B10 alone or 3B10 in combination with either pembrolizumab or Abraxane, highlighting the potential benefit of PTN inhibition alongside immunotherapy and chemotherapy.

## 5. Summary and Perspectives

### 5.1. Integrating PTN Biology Across Development, Tumour Progression, and Subtype Context

**Figure 6:**
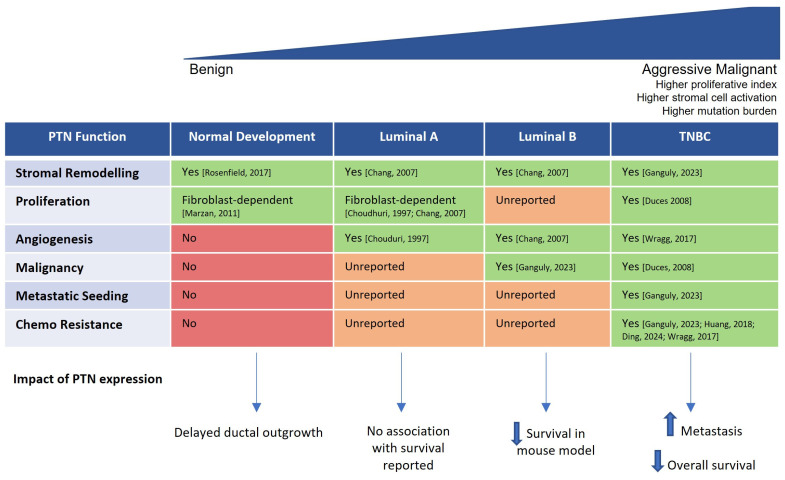
PTN functional landscape across mammary biology and breast cancer subtypes. Summary table illustrating the reported functions of pleiotrophin across normal mammary development, luminal A, luminal B, and triple-negative breast cancer (TNBC). Rows list major PTN-associated processes—stromal remodelling, proliferation, angiogenesis, malignancy, metastatic seeding, and chemotherapy resistance—and each cell is colour-coded according to the strength of published evidence (green = function reported, salmon = unreported function, red = no functional role reported), with references indicated in-cell. A wedge-shaped header depicts the progression from benign to aggressive malignant biology, highlighting increasing proliferation index, stromal activation, and mutation burden from left to right. Beneath the table, arrows summarise the impact of PTN expression in each context: delayed ductal outgrowth in normal development; no reported association with survival in luminal A tumours; decreased survival in a mouse model of luminal B disease; and increased metastasis with reduced overall survival in TNBC. This integrated visual framework emphasises the context-dependent nature of PTN biology and its selective functional activation in aggressive breast cancer [3,21,33,35,59,63,67,68,69].

Pleiotrophin emerges as a context-dependent regulator whose function differs markedly between normal mammary development and breast cancer, as summarised in Figure 6. Artificial Intelligence-generated summaries of its role in breast cancer are quick to summarise that PTN is highly expressed in breast cancer, particularly in more aggressive subtypes. However, as we have seen here, the data gathered to date is considerably more nuanced.

During normal mammary development, PTN expression is relatively high and appears to act as a developmental brake, restraining ductal outgrowth. This effect likely reflects modulation of epithelial–extracellular matrix interactions rather than direct suppression of proliferation. In contrast, in breast cancer models, PTN acquires tumour-promoting functions, including enhanced proliferation, migration, angiogenesis, stromal remodelling, and metastatic colonisation. Understanding how PTN shifts from a developmental regulator to a tumour promoting factor remains an important unresolved question.

A major complexity is the discrepancy between RNA and protein expression. Transcriptomic datasets consistently show higher *PTN* RNA in normal breast tissue than in breast cancer, and within breast cancer the highest expression occurs in good prognosis tumours such as luminal A and normal-like subtypes. Yet available protein level studies suggest that PTN protein is elevated in breast tumours compared with normal tissue. This mismatch raises the possibility of post-transcriptional regulation, altered secretion, differences in protein stability, or substantial paracrine influences and stromal contributions, as summarised in Figure 5. The contribution of adipose tissue should also not be overlooked. Transcriptomic data indicates that *Ptn* is expressed in mouse mammary adipocytes although direct comparisons with other cell types are not yet available. Given the established paracrine influence of mammary adipose tissue, particularly in obesity-associated settings, adipocyte-derived PTN may represent an additional source of signalling within the local microenvironment. Resolving this discrepancy will require systematic proteomic and spatial analyses across breast cancer subtypes.

The functional consequences of PTN expression also appear to be subtype specific. Although luminal A tumours express higher *PTN* RNA, *PTN* expression does not correlate with poor survival in this subtype (Figure 4). In contrast, high *PTN* expression is associated with significantly worse overall survival in basal-like breast cancer. This divergence suggests that PTN’s impact depends on the cellular and microenvironmental context, including receptor availability, stromal composition, and the broader signalling environment. PTN interacts with multiple receptors, including PTPRZ1, ALK, syndecans, integrins, and nucleolin, activating downstream Src/FAK, MAPK/ERK, and PI3K–AKT signalling pathways, providing a mechanistic basis for context-specific signalling outputs. The impact of PTN’s function may only manifest in tumours that are already primed for these processes. For example, increased angiogenesis is likely to have limited impact in luminal tumours with low migratory potential, but becomes far more consequential in TNBC, where a pro-metastatic environment is already established. Because PTN is secreted, its effects often depend on the surrounding cells rather than the tumour cells alone. Several studies show that PTN produced by fibroblasts, endothelial cells, or other stromal components can influence tumour behaviour. This aligns with broader models of tumour–stroma communication in which fibroblasts, endothelial cells, immune cells and ECM components collectively shape tumour progression through reciprocal signalling interactions [70]. This perspective helps explain why PTN has strong effects in subtypes with highly activated stroma, such as TNBC, but weaker or absent effects in luminal A tumours where stromal activation is limited. It also highlights that PTN’s impact reflects interactions within the wider tissue environment rather than tumour cell expression alone.

Taken together, these findings depict PTN as a multifaceted, context-dependent regulator whose biological effects depend on tissue state, cellular origin, breast cancer subtype, and the balance between autocrine and paracrine signalling. Clarifying these relationships will be essential for determining which patients, which tumour contexts, and which PTN dependent processes are most relevant for therapeutic targeting or biomarker development. The limited exploration of PTN in luminal or Her2 cancers and the lack of subtype resolved proteomic data represent important opportunities for future investigation.

### 5.2. Clinical Implications and Future Directions

Across multiple cancer types, PTN has emerged as a clinically relevant molecule with potential value both as a biomarker and as a therapeutic target. A recent meta-analysis of immunohistochemistry-based studies across nine tumour types reported that high PTN protein expression in tumour tissue was consistently associated with advanced stage and poor overall survival, suggesting that PTN may serve as a useful indicator of unfavourable prognosis [71]. With only one breast cancer study included in this analysis, the broader pattern across cancers supports the idea that PTN contributes to clinically meaningful aspects of tumour progression and may be a potential target for tumour treatments [71].

Serum PTN has also shown promise as a minimally invasive biomarker. In breast cancer, elevated circulating PTN correlates with disease presence and poorer survival outcomes, and PTN has been reported to outperform established markers such as CEA and CA15-3 [1]. Similar associations have been observed for PTN in other tumour types, including pancreatic, colon, prostate, multiple myeloma and lung cancer [72,73,74,75,76,77]. However, this pattern is not universal; for example, no association was detected between serum PTN and stomach cancer [72]. Interestingly, for several cancers in which serum PTN is elevated, TCGA data indicate lower *PTN* RNA expression in tumour tissue compared with matched normal tissue, suggesting that circulating PTN may arise from stromal or systemic sources rather than tumour cells alone. This mirrors the uncertainty surrounding the origin of serum PTN in breast cancer and highlights the need for studies that integrate tissue, stromal and circulating measurements. In an era where multi-omics signatures can more accurately capture tumour heterogeneity than standalone biomarkers, PTN remains noteworthy because it reflects not only tumour cell activity but also the broader signalling and communication dynamics of the tumour microenvironment [78]. This view aligns with multi-omics studies highlighting that context-dependent oncogenic determinants arise from the integration of genomic, transcriptomic and microenvironmental signals [79]. Importantly, the clinical utility of serum PTN will depend on assay standardisation and validation in larger, well characterised patient cohorts to determine its robustness, specificity, and added value over existing biomarkers.

Therapeutically, targeting PTN is gaining traction as a potential strategy to slow tumour progression and enhance the efficacy of existing treatments. Experimental approaches such as the delivery of truncated PTN have demonstrated reduced tumour formation in breast cancer models [63]. Other studies show that reducing PTN levels can enhance responses to chemotherapy, immunotherapy, anti-FAK agents and anti-angiogenic therapies. Recent work in ovarian cancer further illustrates this potential: Tenacissoside G, a component of a traditional Chinese medicine (*Marsdenia tenacissima*) that is used clinically to support chemotherapy, was shown to inhibit PTN signalling and reverse paclitaxel resistance in vitro in an ovarian cancer cell line [80]. These finding suggest that PTN-directed therapies may not be as distant from clinical application as previously assumed, and that PTN could represent a valuable point of intervention across multiple cancer types.

Given the breadth of associations between PTN and tumour progression, future work should prioritise identifying the patient groups most likely to benefit from PTN-based biomarkers or therapies. In breast cancer, this will require subtype specific analyses, particularly in luminal tumours where PTN biology remains underexplored, and in TNBC where PTN appears most strongly linked to aggressive behaviour. Integrating proteomic, spatial, and circulating PTN measurements will be essential for clarifying the cellular origin of PTN in different contexts and for determining how best to incorporate PTN into clinical decision-making.

### 5.3. Concluding Remarks

PTN sits at the intersection of development, tumour progression, and microenvironmental signalling, and its impact varies markedly across breast cancer subtypes. What emerges from the available evidence is not a single unifying role, but a context-dependent molecule whose effects depend on tissue state, cellular origin, and the surrounding stromal environment. Clarifying these relationships will be essential for determining when PTN acts as a driver of disease and when it is simply a marker of underlying biology. Continued integration of molecular, spatial, and clinical data will be key to defining PTN’s true potential as both a biomarker and a therapeutic target.

## Data Availability

No new data were created or analysed in this study.
